# Can Melatonin Act as an Antioxidant in Hydrogen Peroxide-Induced Oxidative Stress Model in Human Peripheral Blood Mononuclear Cells?

**DOI:** 10.1155/2016/5857940

**Published:** 2016-01-11

**Authors:** Solaleh Emamgholipour, Arash Hossein-Nezhad, Mohammad Ansari

**Affiliations:** ^1^Department of Clinical Biochemistry, School of Medicine, Tehran University of Medical Sciences, P.O. Box 14155-6447, Tehran, Iran; ^2^Department of Medicine, Section of Endocrinology, Nutrition, and Diabetes, Vitamin D, Skin and Bone Research Laboratory, Boston University Medical Center, 85 E. Newton Street Fuller Building, Boston, MA 02118, USA; ^3^Osteoporosis Research Center, Endocrinology and Metabolism Clinical Sciences Institute, Tehran University of Medical Sciences, P.O. Box 1411413137, Tehran, Iran

## Abstract

*Purpose.* We aimed to investigate the possible effects of melatonin on gene expressions and activities of MnSOD and catalase under conditions of oxidative stress induced by hydrogen peroxide (H_2_O_2_) in peripheral blood mononuclear cells (PBMCs).* Materials and Methods.* PBMCs were isolated from healthy subjects and treated as follows: (1) control (only with 0.1% DMSO for 12 h); (2) melatonin (1 mM) for 12 h; (3) H_2_O_2_ (250 *μ*M) for 2 h; (4) H_2_O_2_ (250 *μ*M) for 2 h following 10 h pretreatment with melatonin (1 mM). The gene expression was evaluated by real-time PCR. MnSOD and catalase activities in PBMCs were determined by colorimetric assays.* Results.* Pretreatment of PBMCs with melatonin significantly augmented expression and activity of MnSOD which were diminished by H_2_O_2_. Melatonin treatment of PBMCs caused a significant upregulation of catalase by almost 2-fold in comparison with untreated cells. However, activity and expression of catalase increased by 1.5-fold in PBMCs under H_2_O_2_-induced oxidative stress compared with untreated cell. Moreover, pretreatment of PBMCs with melatonin resulted in a significant 1.8-fold increase in catalase expression compared to PBMCs treated only with H_2_O_2_.* Conclusion.* It seems that melatonin could prevent from undesirable impacts of H_2_O_2_-induced oxidative stress on MnSOD downregulation. Moreover, melatonin could promote inductive effect of H_2_O_2_ on catalase mRNA expression.

## 1. Introduction

Oxidative stress resulting from an imbalance between reactive oxygen species (ROS) production and neutralization plays an important role in the development of multiple pathophysiological states such as diabetes, atherosclerosis, cancers, neurodegenerative diseases, aging, and autoimmunity. In these conditions, antioxidant defense system is also impaired and subsequently increases oxidative burden [1–4].

Under aerobic situations, ROS are produced as a result of cellular metabolism (e.g., during electron transport in the mitochondria and in the endoplasmic reticulum), function of several oxidase enzymes (e.g., NADPH oxidases, xanthine oxidase, and cytochrome p450 oxidase), and metal-catalyzed oxidative reactions. In normal situations, ROS participates in regulation of normal cellular functions. However, if excess ROS are not effectively eliminated by antioxidant defense system, it causes cellular damage by influencing gene expression and by oxidating DNA, proteins, and lipids. Antioxidant defense systems, both enzymatic and nonenzymatic, keep ROS in check and protect tissue from oxidative damage. Superoxide dismutases (SODs) including MnSOD, Cu/Zn-SOD, and extracellular SOD along with catalase are composed of the first line of defense against ROS. MnSOD is found in the mitochondrial matrix which dismutates superoxide to hydrogen peroxide (H_2_O_2_). Catalase is localized in peroxisomes, mitochondria, nucleus, and sarcoplasms and converts H_2_O_2_ to water and oxygen [5–8]. Deficiencies in MnSOD and catalase are associated with development of a broad range of metabolic disorders, inflammatory conditions, autoimmunity, and cancers. There is also evidence that activation of antioxidant enzymes improves these conditions [9–11].

In the last decade, melatonin (N-acetyl-5-methoxytryptamine) has received a great deal of attention as a potential therapeutic strategy for the prevention of stress-related disorders. Melatonin is a pleiotropic hormone mainly secreted by pineal gland. Melatonin treatment has been also found to exert beneficial effects in metabolic disorders via regulating body weight, energy expenditure, and lipid and glucose metabolism [12]. This hormone also regulates circadian rhythms, inflammation pathways, and apoptosis. However, melatonin is known for its effects on oxidative stress pathways [8, 13]. It is evident that melatonin acts as an antioxidant through free radical scavenging properties as well as by its role in activating several antioxidant enzymes [14, 15].

Peripheral blood mononuclear cells (PBMCs), a heterogeneous population of immune cells, are helpful in revealing the complex nature of the disease, and in biomonitoring studies [16]. There is evidence that PBMCs are sensitive targets to oxidative stress and can reflect systemic symptoms of oxidative damage [17–19]. Despite evidence reporting the effects of melatonin on components of the cellular oxidative stress defense in various* in vivo* and* in vitro* studies, to our knowledge, no study has investigated the antioxidative effects of melatonin in oxidative stress model in PBMCs. In addition, there is no report on impact of H_2_O_2_-induced oxidative stress on antioxidant enzymes in human PBMCs. In this study, we first investigated the impact of H_2_O_2_-induced oxidative stress on antioxidant enzymes in PBMCs by studying the changes in MnSOD and catalase as two primary antioxidative defense enzymes. Secondly, we investigated the possible effect of melatonin against oxidative stress in human PBMCs.

## 2. Materials and Methods

### 2.1. Subjects

This experimental study was conducted on 14 healthy volunteers with an age between 20 and 40 years. Participants were excluded if they had any of the following: (1) history of malignancy, diabetes (types I and II), acute or chronic infection, and any known systemic disease; (2) medication with antioxidant supplements, anti-inflammatory drugs, and vitamins in the previous 6 months. It should be noted that none of the volunteers was currently smoking. The study protocol was approved by the Ethics Committee of Tehran University of Medical Sciences, and written informed consent was obtained from all subjects.

### 2.2. Isolation of PBMCs

After an overnight fast, peripheral blood sample (30 mL) was obtained from all volunteers and collected in sodium-heparin blood collection tubes. PBMC isolation was performed immediately using Ficoll-Hypaque (Lympholyte-H; Cedarlane Laboratories, Hornby, ON, Canada) gradient centrifugation. Briefly, blood from each subject was diluted with equal volume of phosphate-buffered saline (PBS), placed on Ficoll medium and centrifuged at 600 ×g for 30 min at room temperature. The PBMC layer was collected and washed twice with sterile PBS solution (GIBCO; Invitrogen Laboratories, UK) by centrifugation at 200 ×g for 15 min. The cell viability was assessed by trypan blue exclusion assay.

### 2.3. Cell Culture and Treatment

Freshly isolated PBMCs were suspended in RPMI 1640 Glutamax medium (GIBCO; Invitrogen Laboratories, UK) supplemented with 10% heat-inactivated fetal bovine serum (GIBCO; Invitrogen Laboratories, UK), and 1% penicillin/streptomycin solution (GIBCO; Invitrogen Laboratories, UK). PBMCs were then plated at a density of 2 × 10^6^ cells/well and 4 × 10^6^ cells/well in 12- and 6-well flat-bottom plates, respectively.

After overnight incubation at 37°C with 5% CO_2_, PBMCs were treated as follows:

1. Control group: PBMCs incubated only with 0.1% DMSO for 12 h.
2. Melatonin group: PBMCs treated with 1 mM melatonin for 12 h.
3. H_2_O_2_ group: PBMCs treated with 250 *μ*M H_2_O_2_ for 2 h.
4. Melatonin + H_2_O_2_ group: PBMCs treated with 250 *μ*M H_2_O_2_ for 2 h following 10 h pretreatment with 1 mM melatonin.

Finally, the cells were collected and stored at −80°C for further experiments. It should be mentioned that melatonin was dissolved in DMSO and diluted in RPMI 1640 before use. The final concentration of DMSO in the culture medium was 0.1%.

### 2.4. Evaluation of Cell Viability under Melatonin Treatment

The cell viability was performed using MTT (3-(4,5-dimethylthiazol-2-yl) 2,5-diphenyl tetrazolium bromide) (Sigma-Aldrich, St. Louis, MO, USA) colorimetric assay, which reflects mitochondrial dehydrogenase activity in viable cells.

Briefly, PBMCs (at density 25 × 10^4^/well) were seeded in 96-well plates and cultured in medium containing different concentration of melatonin (1 nM, 1 *μ*M, and 1 mM) and incubated for 12 h at 37°C in the humidified atmosphere with 5% CO_2_. Then, PBMCs were washed tree times with phosphate buffer saline and the MTT labeling reagent (500 *μ*g/mL) was added to wells and PBMCs were further incubated for 4 h at 37°C. The media were discarded and the blue formazan crystals were solubilized with DMSO. The percentage of viable cells was calculated by the following formula: a ratio of absorbance of treated cells to control cells (treated with 0.1% DMSO) at 540 nm.

The MTT assay was also performed to evaluate cell viability in the presence of different concentration of H_2_O_2_ (50 *μ*M, 100 *μ*M, 250 *μ*M, and 500 *μ*M) with the same procedure mentioned above.

### 2.5. Quantitative Real-Time PCR

RNA extraction from PBMCs was performed using a Total RNA Extraction Miniprep kit (Viogene, Taiwan) based on the manufacturer's instruction. The RNA purity and concentration were determined using a NanoDrop spectrophotometer. The RNA quality was checked by agarose gel electrophoresis.

The complementary DNA (cDNA) synthesis was performed on one microgram of DNase-treated RNA using Revert Aid First Strand cDNA Synthesis kit (Thermo Scientific, Fermentas, USA).

Real-time PCR was carried out in a Rotor Gene real-time thermocycler (Qiagen, Hilden, Germany) using SYBR Green detection kit (Takara Bio, Otsu, Japan) according to the manufacturer's protocol. Primers for target genes (MnSOD and catalase) and housekeeping gene, *β*-actin, were purchased from Qiagen (Hilden, Germany). Standard curves were generated for all studied genes to evaluate the linear range of the real-time PCR before performing the assay on test samples. The correlation coefficients of all the standard curves were more than 0.95 indicating a good linearity, and all test samples were verified to be within this range. It should be noted that melting curve analysis and subsequent gel electrophoresis were used to confirm the specificity of PCR products. Relative gene expression was normalized to *β*-actin and calculated as 2^−ΔCT^ using the formula: 2^−(Ct target gene−Ct *β*-actin)^.

### 2.6. The Measurement of MnSOD Activity

MnSOD activity was measured in PBMCs using commercially available kit (SOD activity Enzo Life Sciences, USA). The assay is based on the principle that MnSOD neutralizes superoxide anions produced by the xanthine/xanthine oxidase system and subsequently inhibits the reduction of WST-1 (water soluble tetrazolium salt) to WST-1 formazan. Briefly, PBMCs were harvested, washed with ice-cold 1x PBS, and lysed as described in kit protocol. Preincubation of the cell lysate with 2 mM potassium cyanide (KCN) was performed to inactivate both Cu/Zn-SOD and extracellular SOD.

It is important to note that the linear range of the assay was evaluated by generating a standard curve prior to measuring the MnSOD activity. Specifically, we created a serial dilution of the cell extracts, ranging from 0.5 *μ*g/25 *μ*L to 50 *μ*g/25 *μ*L, with 1x SOD buffer. Afterward, the percentage of inhibition of the formation rate of WST-1 formazan was compared with a standard curve generated with serial dilutions of the SOD standard. The SOD standard, WST-1 reagent, and 1x SOD buffer were supplied in kit form. Protein concentration was determined using Bradford method [20]. MnSOD activity values were expressed as units/mg of protein.

### 2.7. The Measurement of Catalase Activity

Catalase activity in PBMCs (2 × 10^6^ cells/well) was determined using the catalase assay kit (Ab83464; Abcam, Cambridge, MA, USA) as per the manufacturer's instructions. In this assay, catalase decomposes H_2_O_2_ to water and oxygen, and then unconverted H_2_O_2_ reacts with OxiRed probe to produce a product that can be measured at 570 nm. Briefly, harvested cells were lysed in a cold assay buffer (provided with kit) and sonication on ice. Following centrifugation, the supernatant was collected and the protein concentration of the resulting cell lysate was determined using the Bradford assay. The catalase activity in the PBMCs was expressed as units/mg of protein.

### 2.8. Statistical Analysis

All data were analyzed using SPSS 19 (SPSS Inc., Chicago, IL, USA) and presented as mean ± standard error of the mean (SEM). Differences between the groups were done using one way ANOVA followed by the* post hoc* Tukey test to compare all pairs of groups. Comparative CT method [21] was used for analysis of the gene expression. Statistical significance was considered at a *p* value < 0.05, unless noted.

## 3. Results

### 3.1. Effect of Melatonin on Cell Viability in PBMC Cultures

**Figure 1:**
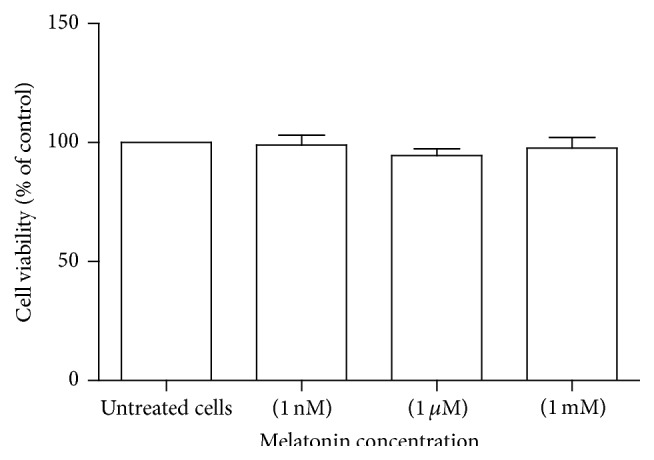
Assessment of PBMCs viability in response to melatonin treatment. Cells were incubated with increasing concentrations of melatonin (1 nM, 1 *μ*M, and 1 mM) for 12 h and cell viability was determined by MTT assay. The MTT assay was performed in triplicate and data are reported from the mean of triplicates. Data are expressed as means ± SEM.

PBMCs were treated with the increasing concentration of melatonin (1 mM, 1 *μ*M, and 1 nM) for 12 h and cell viability was assessed by MTT assay. We observed no significant change in cell viability after exposure to 1 mM (98.9 ± 4.1%), 1 *μ*M (94.6 ± 2.8%), and 1 nM (97.7 ± 4.5%) melatonin in comparison with untreated PBMCs and the cell viability was more than 90% under treatment with these concentrations (Figure 1).

To find the optimum concentration of melatonin for further experiments, we performed a preliminary study, in which melatonin concentrations of 1 mM, 1 *μ*M, and 1 nM were evaluated for their effect on MnSOD and catalase mRNA expression. Our results showed that melatonin concentration of 1 mM had the highest effect on mRNA expression (data not shown). Accordingly, we selected treatment with melatonin at a concentration of 1 mM for next experiments.

### 3.2. Effect of H_2_O_2_ on Cell Viability in PBMCs Cultures

**Figure 2:**
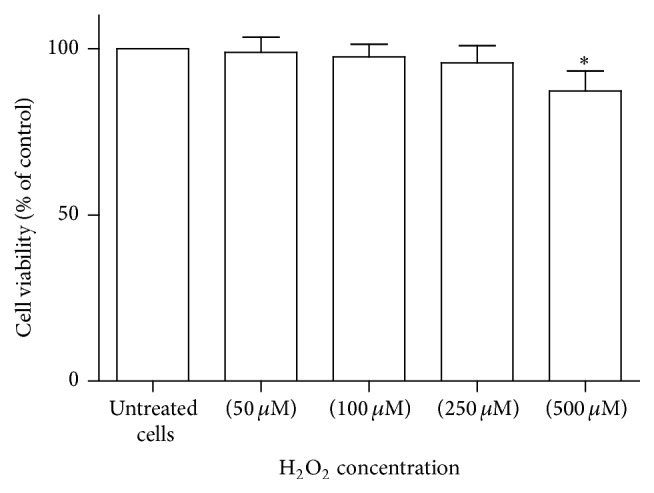
Assessment of PBMCs viability in response to H_2_O_2_ treatment. Cells were incubated with increasing concentrations of H_2_O_2_ (50 *μ*M, 100 *μ*M, 250 *μ*M, and 500 *μ*M) for 2 h and cell viability was determined by MTT assay. The MTT assay was performed in triplicate and data are reported from the mean of triplicates. Data are expressed as means ± SEM. ^*∗*^ *p* < 0.05.

To evaluate the influence of H_2_O_2_ treatment on cell viability, PBMCs were treated with increasing concentration of H_2_O_2_ (50 *μ*M, 100 *μ*M, 250 *μ*M, and 500 *μ*M) for 2 h and MTT assay was performed. The treatment of PBMCs with H_2_O_2_ at concentration of 50 *μ*M, 100 *μ*M, and 250 *μ*M showed no cytotoxic effect on PBMCs and cell viability was more than 90% under PBMCs treatment with these concentrations. However, the exposure of PBMCs with H_2_O_2_ at 500 *μ*M for 2 h significantly diminished the cell viability to 87% (*p* = 0.02) (Figure 2).

To induce oxidative stress for further experiments, H_2_O_2_ at concentration of 250 *μ*M was selected as we found no significant decrease in cell viability in this concentration. Additionally, based on preliminary data, alteration of MnSOD and catalase gene expression was the most pronounced at this concentration compared with 50 *μ*M and 100 *μ*M of H_2_O_2_.

### 3.3. The Assessment of mRNA Expression and Activity of MnSOD under Treatment of PBMCs with Melatonin, H_2_O_2_, and Melatonin plus H_2_O_2_

**Figure 3:**
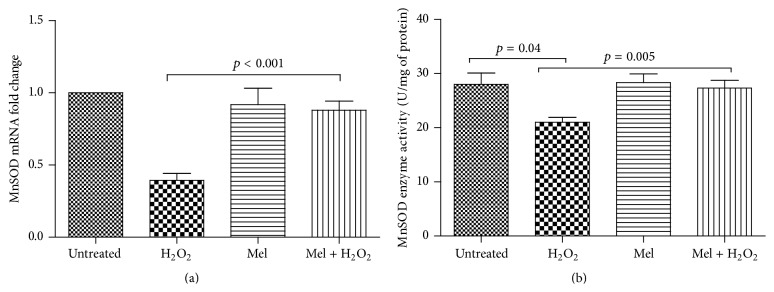
Effect of melatonin, H_2_O_2_, and melatonin plus H_2_O_2_ on (a) mRNA expression and (b) enzyme activity of MnSOD in PBMCs from healthy subjects. PBMCs were treated as follows: untreated (only with 0.1% DMSO for 12 h); melatonin (1 mM) for 12 h; H_2_O_2_ (250 *μ*M) for 2 h; and melatonin plus H_2_O_2_ group receiving H_2_O_2_ (250 *μ*M) for 2 h following 10 h pretreatment with melatonin (1 mM). All untreated cells were treated with 0.1% of DMSO as a control. Data are expressed as mean ± SEM.

We found no alteration in mRNA expression and enzyme activity of MnSOD in PBMCs treated with 1 mM melatonin compared to untreated cells (Figures 3(a) and 3(b)).

To address the possible effect of oxidative stress on expression and enzyme activity of antioxidant enzymes, PBMCs were treated with 250 *μ*M H_2_O_2_ for 2 h. Treatment of PBMCs with 250 *μ*M H_2_O_2_ resulted in a 2.5-fold decrease in MnSOD mRNA levels (Figure 3(a)), in comparison with untreated cell, although it was not statistically significant (*p* = 0.19) (Figure 3(a)). Moreover, H_2_O_2_-induced oxidative stress significantly diminished MnSOD enzyme activity in PBMCs to 25% of baseline level (*p* = 0.04) (Figure 3(b)).

Interestingly, pretreatment of PBMCs with melatonin (1 mM) prior to exposure to H_2_O_2_ (250 *μ*M) caused a significant 2.2-fold increase in MnSOD mRNA levels in comparison with PBMCs treated only with H_2_O_2_ (*p* < 0.001) (Figure 3(a)). Pretreatment of PBMCs with melatonin before addition of H_2_O_2_ also significantly suppressed the decreasing effect of H_2_O_2_ on MnSOD enzyme activity by 30% (*p* = 0.005) (Figure 3(b)).

### 3.4. The Assessment of mRNA Expression and Activity of Catalase under Treatment of PBMCs with Melatonin, H_2_O_2_, and Melatonin plus H_2_O_2_

**Figure 4:**
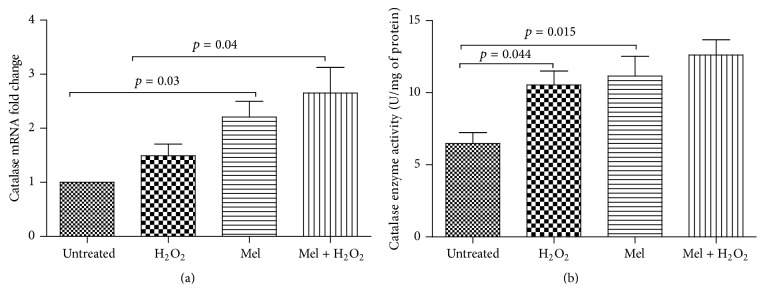
Effect of melatonin, H_2_O_2_, and melatonin plus H_2_O_2_ on (a) mRNA expression and (b) enzyme activity of catalase in PBMCs from healthy subjects. PBMCs were treated as follows: untreated (only with 0.1% DMSO for 12 h); melatonin (1 mM) for 12 h; H_2_O_2_ (250 *μ*M) for 2 h; and melatonin plus H_2_O_2_ group receiving H_2_O_2_ (250 *μ*M) for 2 h following 10 h pretreatment with melatonin (1 mM). All untreated cells were treated with 0.1% of DMSO as a control. Data are expressed as mean ± SEM.

Melatonin treatment of PBMCs resulted in an almost 2.2-fold increase in catalase mRNA expression (*p* = 0.03) (Figure 4(a)), in comparison with untreated cells. With regard to catalase activity, an approximately 80% increase was found in PBMCs treated with melatonin (1 mM) in comparison with untreated cells (*p* = 0.015) (Figure 4(b)).

Catalase mRNA expression increased by 1.5-fold in PBMCs under H_2_O_2_-induced oxidative stress compared with untreated cell although it was not statistically significant (*p* = 0.6). Moreover, H_2_O_2_-induced oxidative stress significantly (*p* = 0.044) elevated catalase enzyme activity in PBMCs compared with untreated cell. Melatonin treatment (1 mM) prior to PBMCs exposure to H_2_O_2_ (250 *μ*M) caused a significant 1.8-fold increase in catalase mRNA levels in comparison with PBMCs treated only with H_2_O_2_ (*p* < 0.001) (Figure 3(a)). Moreover, melatonin treatment of PBMCs before addition of H_2_O_2_ nonsignificantly (*p* = 0.5) enhanced the inductive effect of oxidative stress on catalase enzyme activity (Figure 4(b)).

## 4. Discussion

The role of melatonin in regulating antioxidant defense system and study on its protective effects against oxidative insult have been identified in various* in vivo* and* in vitro* studies [8, 15]. There is evidence that PBMCs are helpful tools to study the status of antioxidant defense system [17–19]. In this study, we examined, for the first time, the melatonin effect against oxidative stress in human PBMCs. In our model system, PBMCs were treated with H_2_O_2_ as a mediator of oxidative stress.

It is well established that high amount of oxidative stress overwhelms antioxidant defense machinery resulting in oxidative injury [22, 23]. In line with this notion, we found a marked decrease in mRNA expression and enzyme activity of MnSOD in H_2_O_2_ treated PBMCs as compared with untreated cells, suggesting the deleterious impacts of oxidative stress on the antioxidant system in PBMC.

However, the catalase mRNA expression and activity were increased following PBMCs exposure to oxidative stress. This observation reflects an adaptive response and a compensatory mechanism for the PBMCs against oxidative stress. This observation corroborates several lines of evidence indicating that cells respond to oxidant challenge with selective induction of certain antioxidant enzymes. In line with this notion, exposure to H_2_O_2_ caused an increase in catalase levels and activity in rat astroglial cells, in human retinal pigment epithelial cells, in hamster tracheal epithelial cells, and in hepatoma cells [24–26]. Some studies also showed that oxidative stress downregulated MnSOD in several oxidative stress models induced by H_2_O_2_ and other oxidants [27, 28]. However, other studies have reported differential regulation of antioxidant enzymes in exposure to oxidative stress [24, 29].

Under increased oxidative stress conditions, peroxynitrite, as a potent reactive oxidant, is produced by reaction between nitric oxide and superoxide anion. It was found that peroxynitrite severely diminishes MnSOD activity by tyrosine nitration both* in vitro* and* in vivo* [30]. Additionally, treatment of rat PBMCs with 100 *μ*M H_2_O_2_ for 1 h caused an accumulation of intracellular superoxide radicals [17]. It is also evident that oxidative addition of superoxide anions to Mn(II)SOD inhibits MnSOD activity through conformational change [31]. Accordingly, the reduced activity of MnSOD under oxidative stress conditions may be related to posttranslational modification of MnSOD and likely its impaired transcriptional regulation. However, further studies are required to identify the responsible mechanism(s).

As summarized above, there is still no general consensus among literature about pattern of antioxidant enzymes in response to oxidative stress. It may be related to the type of cellular model used, the type and concentration of oxidant agent, and incubation time.

Oxidative stress-induced downregulation of MnSOD activity causes superoxide radical accumulation and superoxide can be converted to hydroxyl radicals in the presence of transition metals (e.g., Fenton reaction). Hydroxyl radical is the most damaging ROS produced by cells [6, 32, 33]. Hence, targeting MnSOD in conjugation with catalase as a well-known H_2_O_2_ removing enzyme is a potential approach for the treatment of oxidative stress-related conditions.

Interestingly, in our study, melatonin significantly reversed H_2_O_2_-induced downregulation of MnSOD to normal levels in PBMCs. Moreover, melatonin pretreatment promotes the inductive effect of oxidative stress on catalase expression. It would permit a countervailing and compensatory mechanism against oxidative challenge.

It was shown that treatment of 3T3-1L preadipocytes with melatonin (1 mM) increased activity of catalase and MnSOD [34]; however, Mayo et al. found no change in mRNA levels for antioxidant enzymes, Cu/Zn-SOD, MnSOD, and glutathione peroxidase in nondifferentiated PC12 cells and the human neuroblastoma cells after treatment with 0.1 mM melatonin [35]. Furthermore, based on a more recent study, melatonin alleviated impaired antioxidant enzyme activity in erythrocytes of multiple sclerosis patients [36].

There is evidence that melatonin administration preserved antioxidant status and SOD to normal levels in alloxan-induced oxidative insult in rats [37]. Our findings also corroborate the study of Dzięgiel et al. in which melatonin induced activity of SOD and catalase in myocardial cells of rats in the course of oxidative stress induced by doxorubicin. They found that activities of glutathione peroxidase, SOD, and catalase were higher in rats receiving doxorubicin and/or doxorubicin plus melatonin than in controls. Treatment of rats with doxorubicin plus melatonin markedly increased catalase activity in comparison with rats intoxicated with doxorubicin alone [38].

Although the exact mechanism in which melatonin regulates antioxidant enzymes has not been recognized, several possibilities derived from experimental studies should be considered. Although pharmacological concentrations of melatonin (more than nanomolar concentration) stimulate gene expression and activity of antioxidant enzymes, these quantities of melatonin may act as a direct radical scavenger. This, in turn, alters the cellular redox state and subsequently regulates antioxidant enzyme activity and/or gene expression [15, 39].

It is well established that melatonin exerts many of its roles by its interaction with membrane receptors (MT1 and MT2) and with RZR/ROR receptors which are located in the nucleus. The existence of melatonin receptors is reported in immune cell including lymphocytes, monocytes, regulatory cells, and peripheral blood nuclear cells in humans. It has been proposed that melatonin regulates antioxidant enzyme activity via interacting with calmodulin, which in turn inactivates RORa transcriptional activity and increments antioxidant enzyme activity and expression. Melatonin also restrains RORa constitutive activity through MT1 receptor [40, 41].

It is proposed that melatonin suppresses the degradation of the nuclear factor-E2-related factor (Nrf2) and increases its nuclear translocation via its interacting with ubiquitin ligase. In the nucleus, Nrf2 stimulates the transcription of antioxidant enzymes (e.g., MnSOD and catalase) through binding to the antioxidant response element (ARE) [42]. However further experimental studies are needed to unravel the related molecular mechanism.

Accordingly, the present study can partly open a new window into the protective effect of melatonin on impaired oxidative stress defense in PBMCs under oxidative stress challenge. Additionally, due to the cross talk between inflammation and oxidative stress [43], the antioxidant role of melatonin under elevated oxidative stress conditions can indirectly counteract the undesirable impacts of ROS on dysregulation of inflammatory pathways. However, some limitation of this study merits consideration here as we could not thoroughly investigate the effect of melatonin on other antioxidant enzymes.

## 5. Conclusion

It appears that melatonin treatment could alleviate the impaired antioxidant defense system in human PBMCs as indicated by a pronounced upregulation of MnSOD and catalase. However, more investigations are necessary to fully determine the effectiveness of melatonin treatment on oxidative stress-related disorders.
